# Autumn distribution of Bristol Bay red king crab using fishery logbooks

**DOI:** 10.1371/journal.pone.0201190

**Published:** 2018-07-20

**Authors:** Leah Sloan Zacher, Gordon H. Kruse, Sarah Mincks Hardy

**Affiliations:** 1 College of Fisheries and Ocean Sciences, University of Alaska Fairbanks, Fairbanks, Alaska, United States of America; 2 College of Fisheries and Ocean Sciences, University of Alaska Fairbanks, Juneau Center, Juneau, Alaska, United States of America; University of Minnesota, UNITED STATES

## Abstract

Spatial distributions of fished species must be well characterized to avoid local depletions, identify critical habitat, and predict and mitigate interactions with other fisheries. The Bristol Bay red king crab (*Paralithodes camtschaticus*) fishery is one of the largest crab fisheries in Alaska. Summer crab distributions have been well documented by decades of bottom trawl surveys. However, crab movement and distribution are poorly understood outside the summer survey period, which creates several management challenges. One important component of fishery management is the existence of no-trawl zones, which are intended to protect crab from bottom trawl fisheries. However, it is difficult to evaluate the placement of no-trawl zones, because most crab bycatch occurs in trawl fisheries during winter when crab distributions are unknown. Daily fishing logs, kept by skippers in the red king crab fleet since 2005, contain detailed information on the spatial distribution of catch and effort of legal sized male crab during the autumn crab fishery. However, data contained in these hand-written logbooks have not been readily accessible. We digitized daily fishing logs from 2005 to 2016 and used spatial information on catch and effort to infer geographic distributions of legal sized male king crab during the crab fishing season. Changes in distribution were tracked across this 12-yr period and comparisons were made between warm and cold temperature regimes. In warm years (2005, 2014–2016), crab aggregated in the center of Bristol Bay, Alaska, while in cold years (2007–2013) they were closer to the Alaska Peninsula. The majority of crab were caught in no-trawl areas (63.4% on average), but variations occurred among years and with temperature regime (40.0–86.8% in no-trawl zones). As temperatures continue to shift in the Bering Sea, it will be important to continue monitoring crab distributions outside the summer survey period.

## Introduction

The exploitation of red king crab (RKC), *Paralithodes camtschaticus*, in Bristol Bay, Alaska, has had a long history, beginning with Japanese harvests in the 1920s [1]. In the late 1960s the domestic fishery greatly expanded, with harvests peaking in 1980 and then rapidly declining over the next few years, resulting in fishery closure in 1983 and again in 1994 and 1995 [2,3]. Since then, Bristol Bay RKC have recovered to smaller yet sustainable stock levels [2] that continue to support one of the most valuable shellfish fisheries in Alaska, with an ex-vessel value ranging over US$62–117 million annually between 2005 and 2014 [4].

The Bristol Bay RKC fishery primarily occurs on the middle shelf of the southeastern Bering Sea, south of 58^o^N and east of 165^o^W at depths between 50 and 100 m. This crab stock is co-managed by the Alaska Department of Fish and Game (ADF&G) and the National Marine Fisheries Service (NMFS), but ADF&G is responsible for day-to-day management under the guidance of the federal king and Tanner crab fishery management plan. Historically, this fishery was managed as “limited entry”, with a guideline harvest level (aka catch quota) set each season for those with permits to participate. The large incentive for vessels to “race for fish” and harvest crab faster than competitors, created many environmental, economic, and safety concerns [5]. In 2005, management changed to a crab rationalization program [6], whereby each harvester is now allocated a percentage (individual fishing quota) of the total allowable catch, and there is no longer a need to “race for fish” because quotas can be harvested any time over the three-month season (October 15 –January 15) [7]. This unique program also allocated processing quota shares, as well as community protections that require a certain portion of the catch to be landed in particular regions [5].

**Fig 1:**
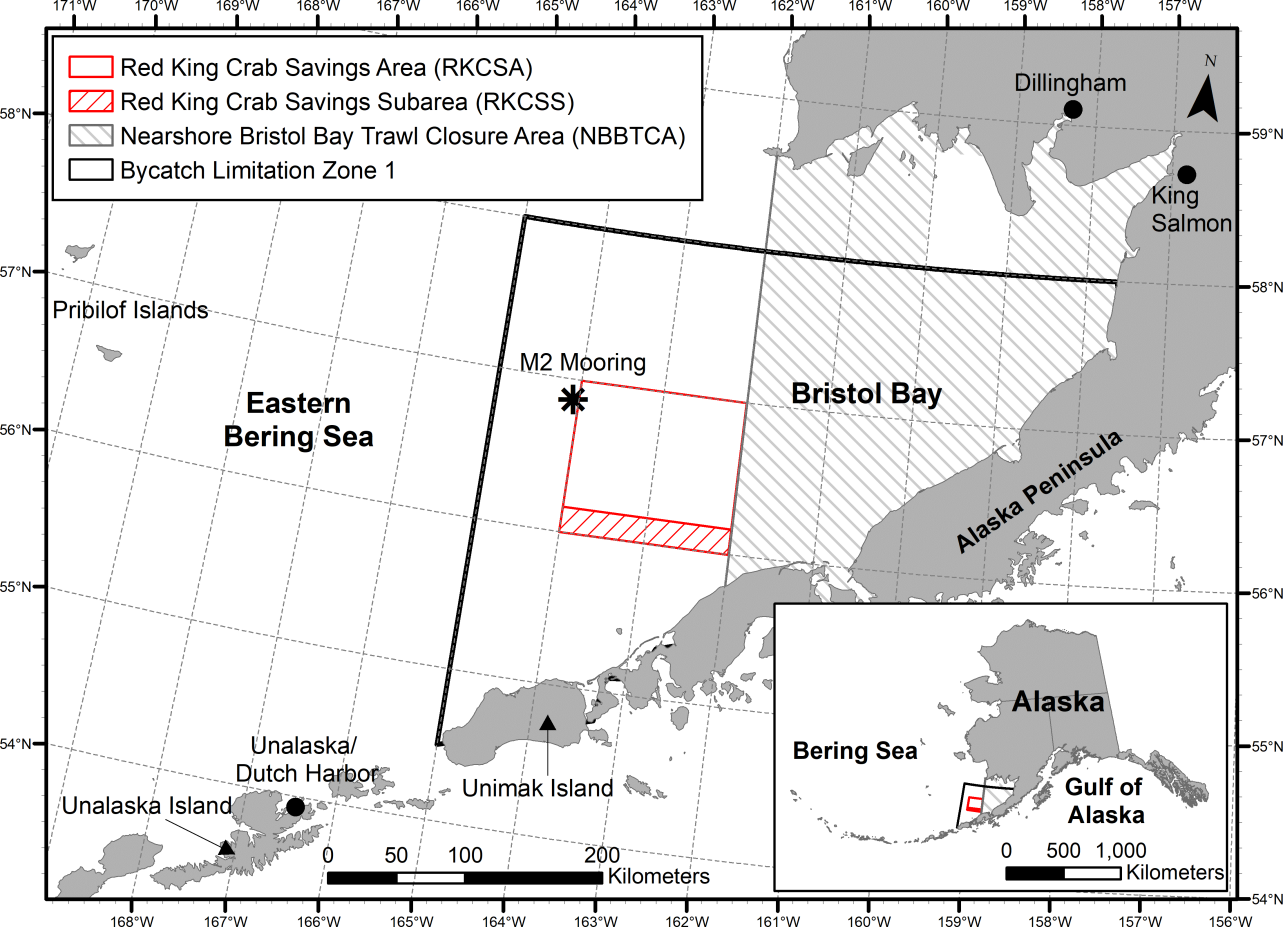
Protected areas for Bristol Bay red king crab in the southeastern Bering Sea. Map shows areas closed to bottom trawling (RKCSA and NBBTCA) and open to trawling, but with bycatch limitations (Zone 1 and RKCSS). Asterisk indicates location of M2 Mooring.

In addition to catch restrictions for the target fishery, Bristol Bay RKC are protected from bycatch in the trawl fisheries through trawl closure areas (Fig 1) and bycatch limits [8]. The Red King Crab Savings Area (RKCSA) was designed to protect adult RKC and prohibits non-pelagic trawls year-round, except in the Red King Crab Savings Sub-Area (RKCSS). Fishing may occur in the sub-area as long as crab abundance in the previous year was high enough to support a directed crab fishery. For the remainder of the manuscript, references to the RKCSA pertain only to the non-trawlable portion, excluding the RKCSS. To protect juvenile RKC, non-pelagic trawling is also prohibited in the Nearshore Bristol Bay Trawl Closure Area (NBBTCA). RKC are a prohibited species in non-pelagic trawl fisheries, meaning that when a designated bycatch limit is reached, all of Zone 1 (Fig 1) is closed to non-pelagic trawling [8].

RKC distributions vary over both seasonal and interannual time scales due to ontogeny, seasonal reproductive cycles, and variable environmental factors. RKC migrate to shallow waters in late winter, and larval release, molting, and mating occur in the spring [9–11]. In the Bering Sea, female RKC typically remain in shallow waters, while males migrate to deeper waters in the late summer and autumn [12]. However, decadal-scale trends in temperature also lead to shifts in distribution of benthic species, including RKC [3,13]. The Bering Sea oscillates between warm and cold temperature regimes, largely driven by sea ice extent [14,15]. In cold years, with greater sea ice extent and later ice retreat in the spring, a pool of cold (< 2 ^o^C) bottom water (called the “cold pool”) persists in the southeastern Bering Sea throughout the summer and into autumn until vertical mixing occurs [14]. In contrast, the cold pool is further north in warm years [13], and bottom waters in the southeastern Bering Sea are several degrees warmer [14]. Over the past 12 years, 2005 and 2014–2016 were warm years, 2006 was a moderate year, and 2007–2013 were cold years [15]. In recent cold years (e.g., 2008, 2009, 2010, 2012), summer distribution of RKC shifted from central Bristol Bay to nearshore regions along the Alaska Peninsula [16]. Shifts in distribution, particularly if unaccounted for in management efforts, are cause for concern, because they may leave crab more vulnerable to habitat disturbance and unintended bycatch [16].

Stock assessments for Bristol Bay RKC are primarily based on the NMFS eastern Bering Sea continental shelf bottom trawl survey for crab and groundfish [17]. This survey has been conducted annually since 1975 in early June through late July/early August, providing an excellent time series with which to examine abundance trends and summer crab distributions. However, no complimentary survey is conducted during the autumn or winter. Thus, our knowledge of autumn/winter crab distributions is based on catch data from the fishing industry collected from fish tickets (landing records), and by onboard observers and port samplers. These data are generally reported by large ADF&G statistical areas (0.5^o^ latitude x 1^o^ of longitude) [18], that do not allow for examination of fishing effort or crab distributions on a finer spatial scale. Data on the spatial distribution of crab bycatch in groundfish fisheries are also available, but only for areas where trawling is permitted. Lack of detailed data on winter RKC distributions is of concern because most RKC bycatch in non-pelagic groundfish trawls occurs in the winter, especially in the rock sole (*Lepidopsetta* spp.) fishery [16,19]. Recognizing the effects of temperature on crab distribution, the North Pacific Fishery Management Council called for an examination of the effectiveness of the current trawl closure areas in Bristol Bay in reducing RKC bycatch [16,20]; however, without detailed data on winter RKC distributions, the effectiveness of trawl closure areas has been difficult to evaluate. This study used catch per unit effort (CPUE) data from fishermen’s logbooks during the autumn RKC fishery to infer crab distributions shortly before the start of winter trawl fisheries.

Daily fishing logs (DFLs), kept by skippers in the RKC fleet since rationalization in 2005, can help improve our understanding of legal sized (≥ 165 mm carapace width) male RKC distributions outside the summer survey period. DFLs contain detailed information on RKC catch and location during the late autumn/early winter fishery, especially for the first month of the fishery when the majority of crab quotas (74%– 100%) are filled (October 15^th^–November 14^th^). DFLs are hand-written by skippers, and carbon copies are submitted to NMFS and ADF&G each year. In addition to DFLs, onboard observer data also provide information on RKC distributions within each fishing season. Since 2005 Bristol Bay RKC observers have recorded detailed data on the catch in sampled pots (aka traps), but they only sample approximately 5% of the pots on 20% of the vessels, equating to ~1–2% of total pots in the fishery annually [18]. Rather than very specific information on just a few pots, DFLs provide an average catch for each pot string (group of ~30 pots) across the entire fishery.

We digitized DFLs from 2005 through 2016 to elucidate the spatial and temporal changes in the winter distribution of legal Bristol Bay RKC. We compared patterns from DFL data to information gained from observer data to determine if these two different sources of information collected from the same fishery yielded similar spatial patterns. Although observer data are more precise for the pots sampled, there are fewer observations, and thus they may not cover the same spatial area. Based on autumn crab distributions from DFLs, we evaluated the effectiveness of the trawl closure areas, and how shifts in RKC distribution relate to large-scale temperature regimes in the Bering Sea.

## Methods and approach

### Daily fishing log data preparation

**Fig 2:**
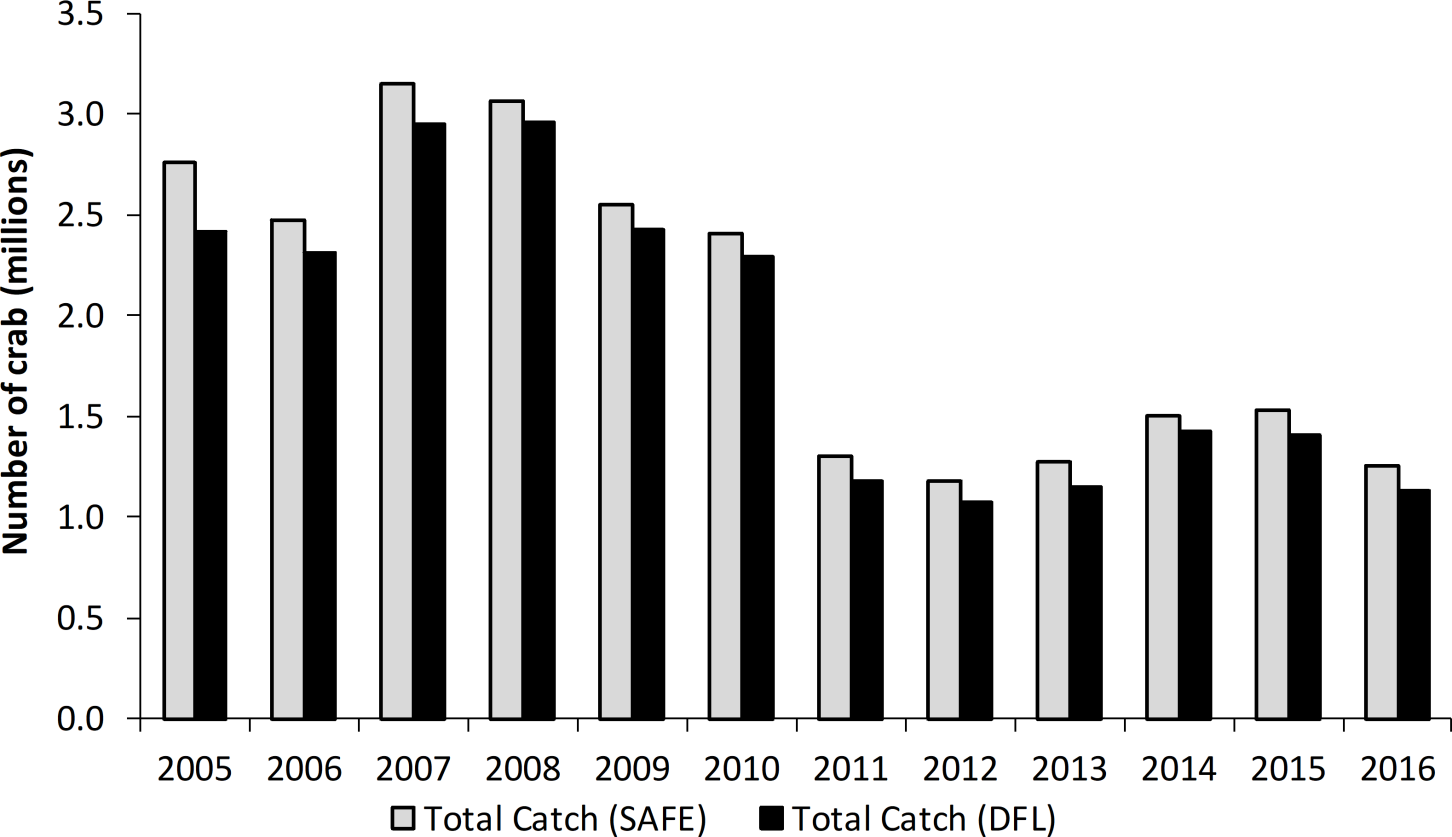
Bristol Bay red king crab catch over 2005–2016. A comparison of catch reported in daily fishing logs (DFLs) and in the crab stock assessment and fishery evaluation (SAFE) report.

DFLs are hand written on carbon paper, with 5 copies of each entry; ADF&G and NMFS each get one copy, while the original stays with the vessel. We used ADF&G copies because archived logs were more accessible and ADF&G copies were expected to be more legible, as they are the second carbon copy, whereas NMFS has the fourth carbon copy. Data from all Bristol Bay RKC DFLs from 2005 through 2016 were entered by hand into spreadsheets, accounting for 29,973 records. Very few records (< 1%) were illegible or incomplete, with an unknown number of DFL pages missing. To evaluate the completeness of these records we compared total catch for each year from DFL records to the fishery records of total catch recorded in the crab stock assessment and fishery evaluation (SAFE) reports [21]. Fig 2 shows that these DFL records encompass a large proportion of the total crab fished each year, from 87.5% in 2005 to 96.6% in 2008.

In the Bristol Bay RKC fishery, crab pots are generally set in straight rows, called strings (29.5 ± 23.9 pots [mean pots per string ± 1 SD]; 10.4 ± 10.2 km [mean string length ± 1 SD]). Each DFL entry represents an entire string, not an individual pot, with the following recorded: coordinates and depth of the first and last pot in each string, date and time pots were set and retrieved, number of pots set and lost, and number and weight of legal RKC caught and retained. Catch per pot was calculated for each DFL string as the number of crab caught divided by the number of pots hauled (pots set minus pots lost). Soak time (63.6 ± 47.3 hours [mean ± 1 SD]) had little effect on catch per pot (R^2^ = 0.05), thus it was not taken into account in these analyses, and nominal catch per pot is used for CPUE.

To eliminate biases from extreme values, data were trimmed in two ways for spatial analyses. First, only strings with > 5 and ≤ 100 pots were included. Strings with only a few pots have a small sample size and are likely to give highly variable values for CPUE, while strings with many pots are unlikely to be set in straight lines and likely cover a larger spatial area than can be understood by the two sets of coordinates provided. In addition, strings with a linear length greater than 20 km were excluded, because they did not provide sufficiently fine spatial resolution. After trimming, records for 26,892 strings remained (90% of total data). The resultant mean string length was reduced to 8.0 ± 3.6 km (±1 SD), and the number of pots per string was reduced to 25.4 ± 12.0 (±1 SD). For each trimmed record, the string midpoint was calculated by averaging the start and end coordinates of the string. The string midpoints were used in all subsequent spatial analyses.

### Spatial analysis

In all spatial analyses, the spatial relationship, or spatial neighborhood, must be defined around each observed value (*i*) of the variable *X* of interest (i.e., CPUE) [22,23]. A matrix of spatial weights (*w*_*ij*_) explains how each value of *i* relates to all other observed values (*j*), and which values (*j*) are included in the neighborhood around each value (*i*) [22]. Spatial weights define the scale of the analysis, and they affect the patterns in autocorrelation across the study area [22,23]. Neighborhood weights can be defined in a variety of ways, including distance (*d*) from *i* or the *k* closest points to *i* [22]. Distances can be fixed, or they can decay with distance from *i* [24]. We tested five different neighborhood weighting methods, including a fixed *d* = 5, 10, and 20 km and *k* = 20 and 40 points (S1 Fig). A *k* of 20 and 40 gave similar results to a *d* of 5 and 10, respectively. However, using a fixed number of neighbors resulted in inherent biases with our data. Data points tended to be sparser in low CPUE areas that fishermen actively avoided, and very dense in high CPUE areas that fishermen targeted. Thus, when *k* was used to define a neighborhood, the low CPUE regions incorporated a greater area around each data point compared with the high CPUE regions. These differences in scale with CPUE made interpretation of results difficult, and we elected to use fixed distance bands, as they provide a uniform scale. Given that point data came from the midpoints of pot strings, and strings were restricted to a maximum of 20 km, the longer strings (>10 km) would not fit within the 5 km distance band. The 20 km distance band was so large that it only gave broad patterns, especially in years when fishing occurred over a relatively small area. A distance band of 10 km gave a finer scale view of spatial relationships, while allowing the entire length of all strings to fall within the 10 km distance band of their midpoints. Therefore, results are shown using the 10 km distance band, as it is the most reasonable for this dataset; sample results with 5 km and 20 km distances are provided in S1 Fig. Given our choice for neighborhood weights, the matrix *w*_*ij*_ is a matrix of zeros and ones. When *j* is within 10 km of *i*, the weight is 1, meaning that *j* is included in the neighborhood of *i*.

Moran’s *I*, Getis-Ord *G*_i_^*^_,_ and local Moran’s *I* statistics were used to identify patterns in the spatial structure of CPUE in string midpoints from the DFL dataset [25–27]. We chose these methods over geostatistical techniques (e.g., kriging) because the goal of this study was to examine patterns and clustering in the point data available, rather than creating a continuous surface through interpolation [28].

Moran’s *I* is a global statistic used to test for spatial autocorrelation in CPUE across the entire study area [26]:

$$I=\frac{n}{\sum_{i=1}^{n}\sum_{j=1}^{n}w_{i,j}}\frac{\sum_{i=1}^{n}\sum_{j=1}^{n}w_{i,j}(x_{i}-\bar{X})(x_{j}-\bar{X})}{\sum_{i=1}^{n}{\left(x_{i}-\bar{X}\right)}^{2}},$$

where $\bar{X}$ is the global mean CPUE, and *x_i_* and *x_j_* are the CPUEs at value *i* and *j*, respectively. A Moran’s *I* > 0 indicates clustering of similar values, *I* < 0 indicates clustering of dissimilar values, and *I* = 0 indicates no autocorrelation or perfect randomness.

The Getis-Ord *G*_i_^*^ statistic was used to measure local spatial clustering and identify areas of high (hot spots) and low (cold spots) CPUE [25]:

$$G_{i}^{*}=\frac{\sum_{j=1}^{n}w_{i,j}x_{j}-\bar{X}\sum_{j=1}^{n}w_{i,j}}{\sqrt{\frac{\sum_{j=1}^{n}x_{j}^{2}}{n}-{\bar{X}}^{2}}\sqrt{\frac{n\sum_{j=1}^{n}w_{i,j}^{2}-{\left(\sum_{j=1}^{n}w_{i,j}\right)}^{2}}{n-1}}}.$$

The *G*_i_* statistic measures positive spatial autocorrelation. The calculation is performed for every data point (string midpoint), *i*, which tests the null hypothesis that the mean of *i* and its neighbors is equal to the global mean. When the null hypothesis is rejected, indicated by a significant *p*-value, *i* is a hot spot when *G*_i_* is positive and a cold spot when *G*_i_* is negative. Due to the nature of this dataset, cold spots are generally data-poor, since fishermen try to avoid these locations. Although the locations of cold spots are shown, we focus our analyses and interpretation on hot spots.

Local Moran’s *I* was used as a secondary local statistic to verify results from the *G*_i_* statistic. This statistic is the local equivalent of the global Moran’s *I*, with:

$$I_{i}=\frac{x_{i}-\bar{X}}{\frac{\sum_{j=1,j\neq i}^{n}w_{i,j}}{n-1}-{\bar{X}}^{2}}\sum_{j=1,j\neq i}^{n}w_{i,j}(x_{i}-\bar{X}).$$

Moran’s *I*_*i*_ measures both positive and negative spatial autocorrelation, thus four significant situations can arise: high-high (*i* and its neighbors are higher than the global mean, similar to *G*_i_* hot spot), low-low (*i* and its neighbors are lower than the global mean, similar to *G*_i_* cold spot), high-low (*i* is a high outlier, with low neighbors), and low-high (*i* is a low outlier, with high neighbors). Unlike the *G*_i_* statistic, Moran’s *I*_*i*_ does not include the value of *i* in the calculation of I_*i*_, which allows for the detection of local outliers (high-low and low-high). We only report the results from the high-high scenario for comparison with the *G*_i_* hot spots. The low-low scenario was poorly resolved, as these low catch areas were already data poor (see above for *G*_i_*) and the elimination of *i* in the calculation of *I*_*i*_ further decreased the data available for calculating *I*_*i*_. Although outliers can prove interesting, they were not the focus of this study and confidentiality restrictions prevent the reporting of point data.

When local autocorrelation analyses are performed in the presence of global autocorrelation, the probability of a Type I error increases, because data points may not be independent, and multiple comparisons are performed [27,29]. However, corrections for multiple comparisons, like Bonferroni, are too conservative, because they assume that all data points are dependent, which is unlikely [23,27]. To be cautious, we defined significance at α = 0.01 and all p-values were calculated using permutations [30]. Observed statistics were compared with reference distributions created from 10,000 permutations of spatially random CPUE data. Pseudo *p*-values were calculated using one-sided significance tests, such that *p = (M + 1)/ (R + 1)*. Here, *R* is the number of permutations and *M* is the number of times the permutated statistic is greater than or equal to the observed statistic (for positive *G*_i_* and *I*_*i*_) or less than or equal to the observed statistic (for negative *G*_i_* and *I*_*i*_) [31].

### Mapping

For each year, two maps were created showing the distribution of *G*_i_^*^ and *I*_*i*_ statistics, first using CPUE data for the entire season, and second using only data from the beginning of the fishery. We defined the “beginning” of the fishery as the first 5% of crab caught out of the total legal crab in the population (hereafter referred to as “first 5%”). Total legal crab abundances in the Bristol Bay RKC population are estimated in the SAFE reports each year; we used estimates from model 2b in the 2017 SAFE [21]. Continued fishing effort drives down the global mean CPUE, and the total allowable catch varies among years, so hot spot analyses are not directly comparable among years when all data are used. The fishing location and catch information of crab vessels is confidential. Therefore, the string midpoint data (all data points, *i*) used in analyses cannot be shown here. To show allowable approximations of these confidential data, we aggregated data points into large irregular polygons. All aggregations were performed using a 10-km aggregation distance, meaning that data points within 10-km of each other were aggregated into the same irregular polygon. Three aggregations were performed for each map. First, all data points, *i*, were aggregated to visualize total fishing extent for each year. Second, all data points, *i*, that were determined to be hot spots were aggregated. Third, all data points, *i*, that were determined to be cold spots were aggregated.

Maps of raw data were also created to show the spatial distribution of CPUE and total catch. A hexagonal grid with 100 km^2^ grid cells was placed over the study area. The CPUE values of all string midpoints falling within a grid cell were averaged and the total crab caught per string were added for all string midpoints in a cell (S1 Table). To adhere to confidentiality requirements, only grid cells containing data from 3 or more vessels are shown.

Hot spots were extracted from each year and all years were overlaid on each other to examine hot spot persistence across years. The same was done for high catch regions (≥ 20,000 crab/grid cell). Because warm and cold years showed different RKC distributions, they were mapped separately. All spatial analyses were conducted in GeoDa version 1.8 [30], while data cleaning, pre-processing, and mapping were done in ArcGIS version 10.3 [32]. Data were projected onto the Alaska Albers Equal Area Conic using the North American 1983 Datum.

### Observer data

Observers report the catch and latitude/longitude coordinates of individual pots, not strings, thus it was not necessary to trim observer data and the entire dataset was used for each year. Across the 12 focal years, observers recorded information on 13,813 pots. Observer data were analyzed using the Moran’s *I* and *G*_i_^*^ statistics and mapped by aggregating points, as described above. Given that the observer dataset has fewer observations than the DFL dataset, cold spots were poorly resolved, thus we only show hot spots here.

## Results

**Fig 3:**
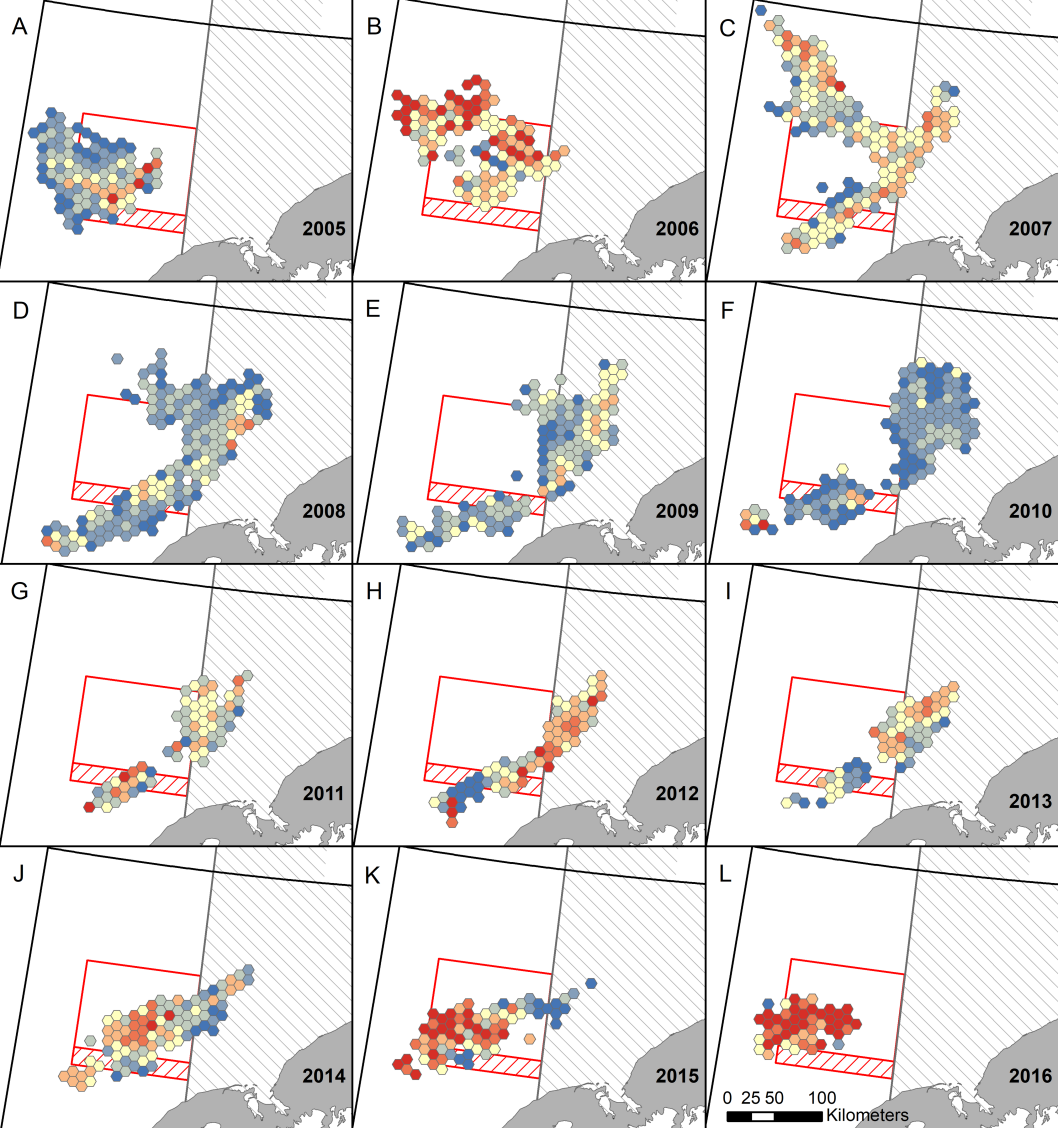
Spatial and temporal distribution of mean catch per unit effort (CPUE). Hexagonal grid cells (100 km^2^) show the mean CPUE of Bristol Bay red king crab using data from daily fishing logs (dark red ≥ 40, medium red = 35–39.9 light red = 30–34.9, cream = 25–29.9, light blue = 20–24.9, medium blue = 15–19.9, and dark blue < 15 mean crab per pot). Only grid cells represented by ≥ 3 vessels are included due to confidentiality restrictions. Areas with restrictions on trawling are outlined in red or gray and are described in Fig 1.

**Fig 4:**
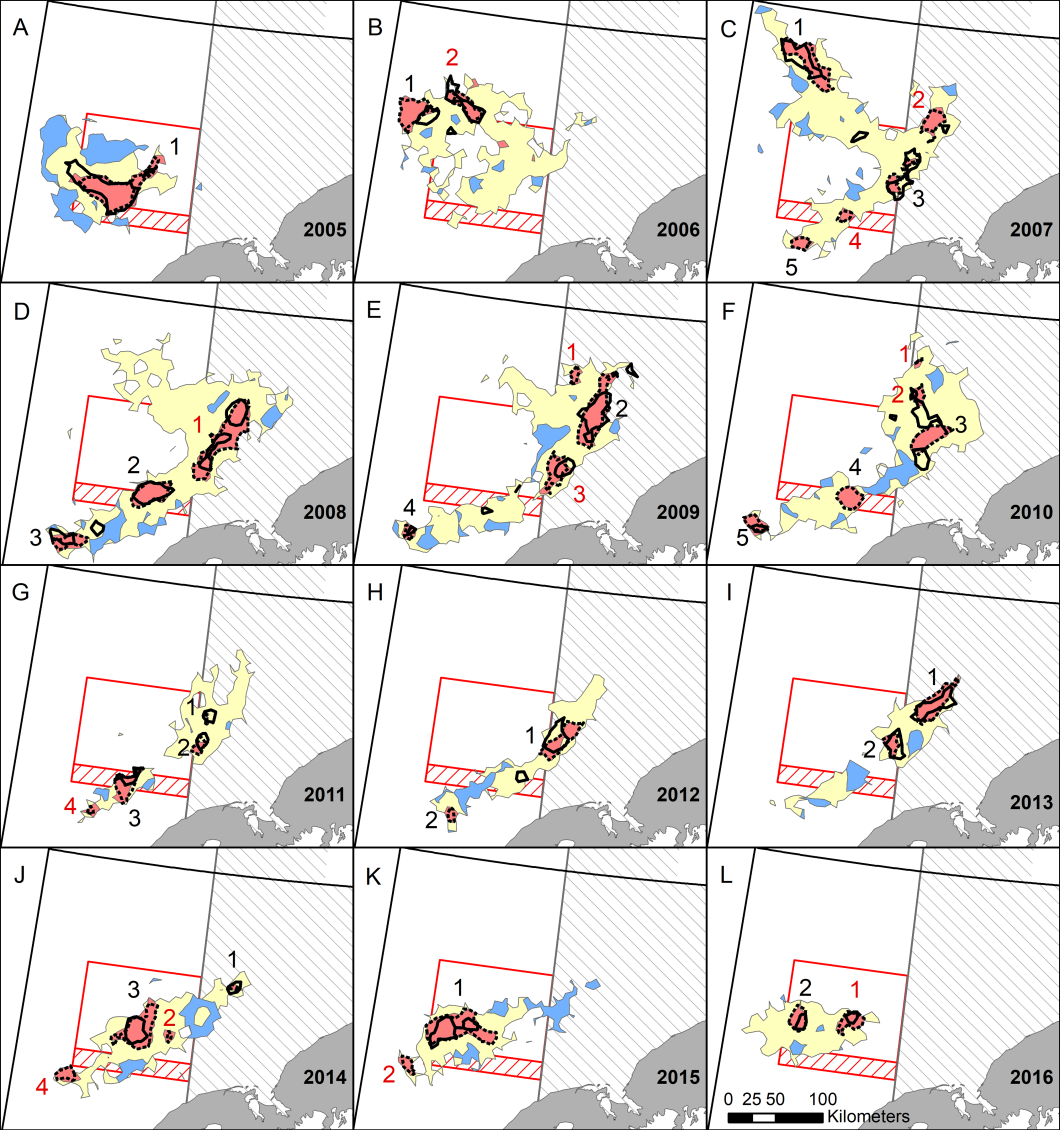
Red king crab distributions using daily fishing logs (DFLs) from the entire season. Red, blue, and yellow areas indicate locations where fishing occurred each fishing season (October 15^th^–January 15^th^). Red areas are detectable hot spots (*G*_i_* indicating statistically significant (*α* < 0.01) high catch per unit effort (CPUE)); blue areas are detectable cold spots (*G*_i_^*^ indicating statistically significant low CPUE); yellow areas indicate locations where CPUE was not statistically different from the mean. Black dashed lines are high-high areas using the local Moran’s *I* statistic on DFL data and solid black lines are hot spots using observer data (*G*_i_^*^ statistic). Hot spots are numbered in color for each year, black when they occur in both the full dataset (this figure) and the first 5% (Fig 5) and red when they occur in just one dataset. Areas with restrictions on trawling are outlined in red or gray and are described in Fig 1.

**Fig 5:**
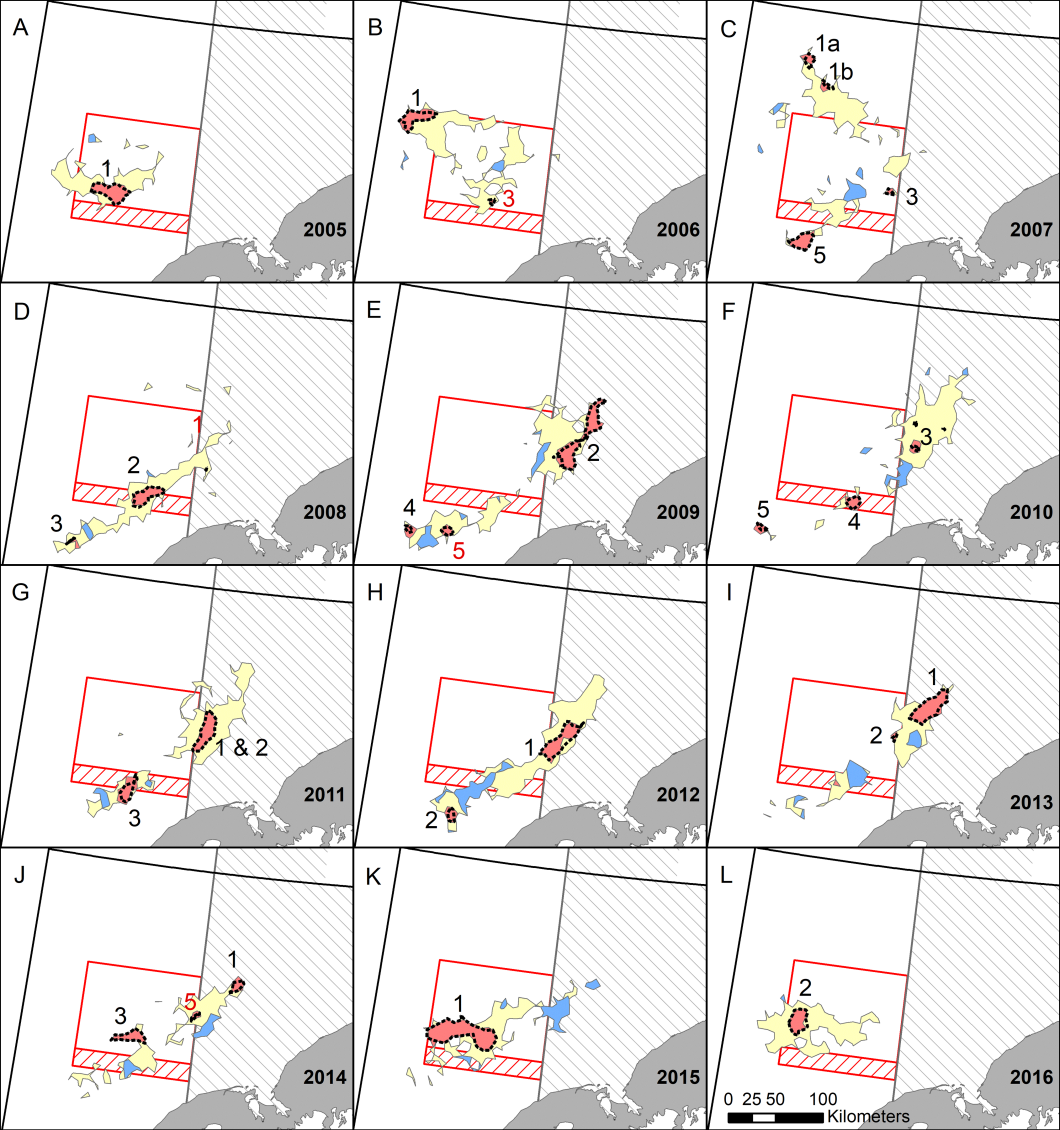
Red king crab distributions using daily fishing logs (DFLs) from the beginning of the season. The beginning of the fishing season (entire season October 15^th^–January 15^th^) was defined as the first 5% of crab caught out of total legal crab in the population. Red, blue, and yellow areas indicate locations where fishing occurred. Red areas are detectable hot spots (Gi* indicating statistically significant *(α* < 0.01) high catch per unit effort (CPUE)); blue areas are detectable cold spots (Gi* indicating statistically significant low CPUE); yellow areas indicate locations where CPUE was not statistically different from the mean. Black dashed lines are high-high areas using the local Moran’s *I* statistic on DFL data and solid black lines are hot spots using observer data (Gi* statistic). Hot spots are numbered in color for each year, black when they occur in both the full dataset (Fig 4) and the first 5% (this figure) and red when they occur in just one dataset. Areas with restrictions on trawling are outlined in red or gray and described in Fig 1.

Based on DFLs from the Bristol Bay RKC fishery, mean CPUE varied from 18 to 40 crab per pot between 2005 and 2016 (S2 Fig), with high variation among years in spatial distribution and intensity of CPUE (Fig 3). On the global scale, positive spatial autocorrelation occurred in all years, when using observer data (*I* = 0.046–0.259; *p* <0.001) and DFL data from the entire fishing season (*I* = 0.090–0.295; *p* < 0.001) and only the first 5% (*I* = 0.124–0.563; *p* < 0.001). On the local scale, statistically significant hot spots and cold spots occurred in all years, in all datasets (Figs 4 and 5).

### Hot spot validation

Hot spots and high-high areas detected using Getis-Ord *G*_i_^*^ and Moran’s *I*_*i*_, respectively, were virtually identical (Fig 4). A few small areas were only detected in one of these analyses (e.g., 2006); henceforth, we only consider hot spots that were also high-high areas. Hot spots detected using observer data generally overlapped with those identified using DFL data, although the degree of correspondence varied among years (Fig 4). In many instances, the locations of DFL and observer hot spots were very similar (e.g., 2005, 2008, 2012), while in other years they did not overlap as much, or some hot spots were only detected in one of the datasets (e.g., 2010, 2011). However, observer hot spots were always near DFL hot spots and never overlapped DFL cold spots.

In general, hot spot maps from the first 5% corresponded well with the entire dataset, with most hot spots being represented in both maps (Figs 4 and 5; black numbered hot spots). However, some hot spots only appeared in one of the maps (Figs 4 and 5; red numbered hot spots). Both maps had the same hot spots in 2005 and 2012, with all other years having at least one difference. For the most part, extra hot spots occurred in the full dataset that did not occur in the first 5%. In some instances, fishing had not yet occurred in these locations in the first 5% dataset (e.g., 2006 hot spot 2, 2007 hot spot 2, 2009 hot spot 3, and 2015 hot spot 2), but in other cases fishing had occurred over at least part of the area, but the area was not considered a hot spot (e.g., 2008 hot spot 1, 2011 hot spot 4, 2016 hot spot 1). In a few years, hot spots occurred in the first 5% dataset, but not in the full dataset (e.g., 2006 hot spot 3, 2009 hot spot 5, 2014 hot spot 5).

### Hot spots versus high catch areas

**Fig 6:**
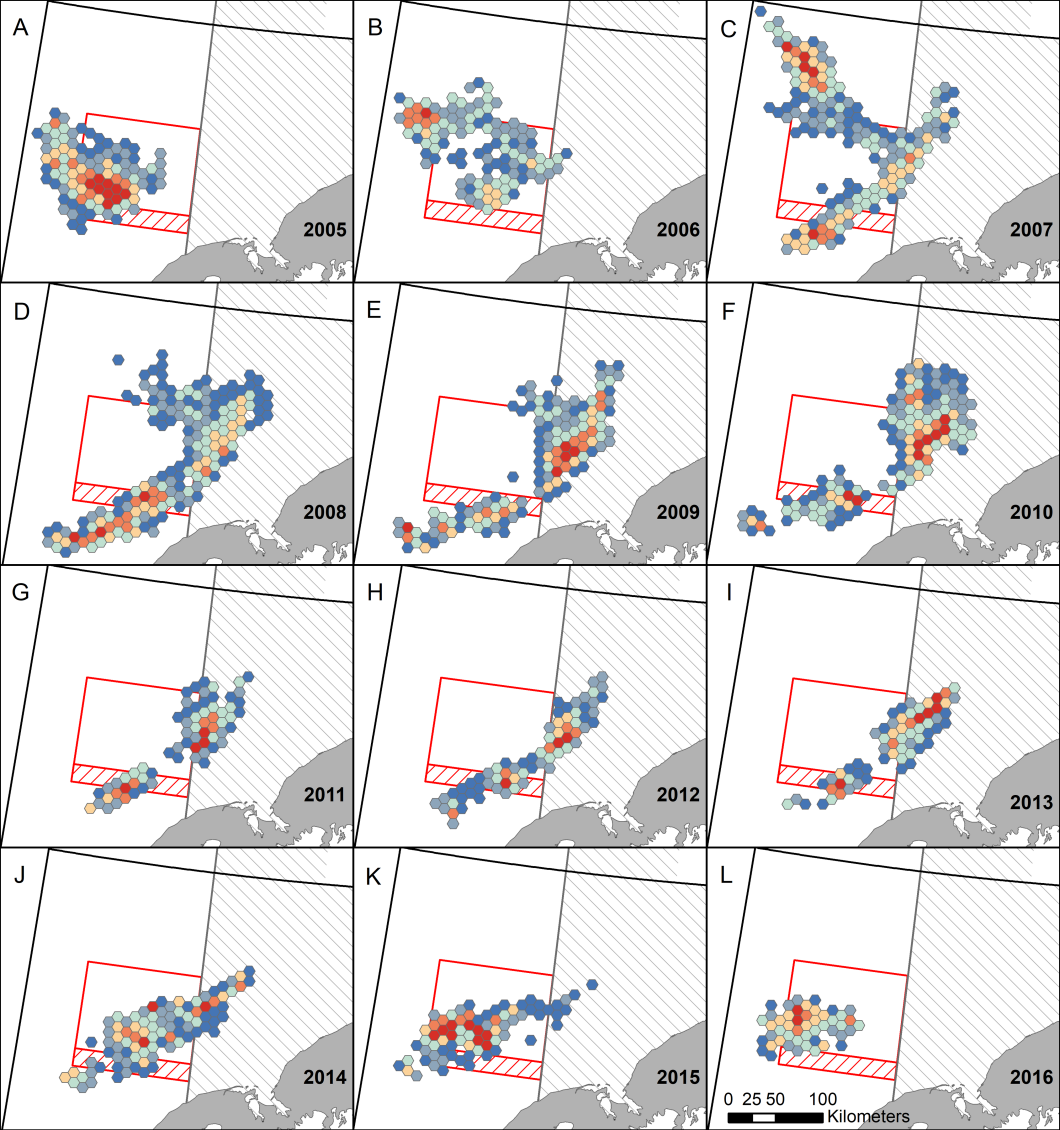
Total catch of legal male red king crab in Bristol Bay using daily fishing logs. Hexagonal grid cells (100 km^2^) show the number of crab caught each fishing season (dark red ≥ 40,000 crab, medium red = 30,000–39,999, light red = 20,000–29,999, light blue = 10,000–19,999, medium blue = 5,000–9,999, and dark blue < 5,000). Only grid cells represented by ≥ 3 vessels are included due to confidentiality restrictions. Areas with restrictions on trawling are outlined in red or gray and are described in Fig 1.

**Fig 7:**
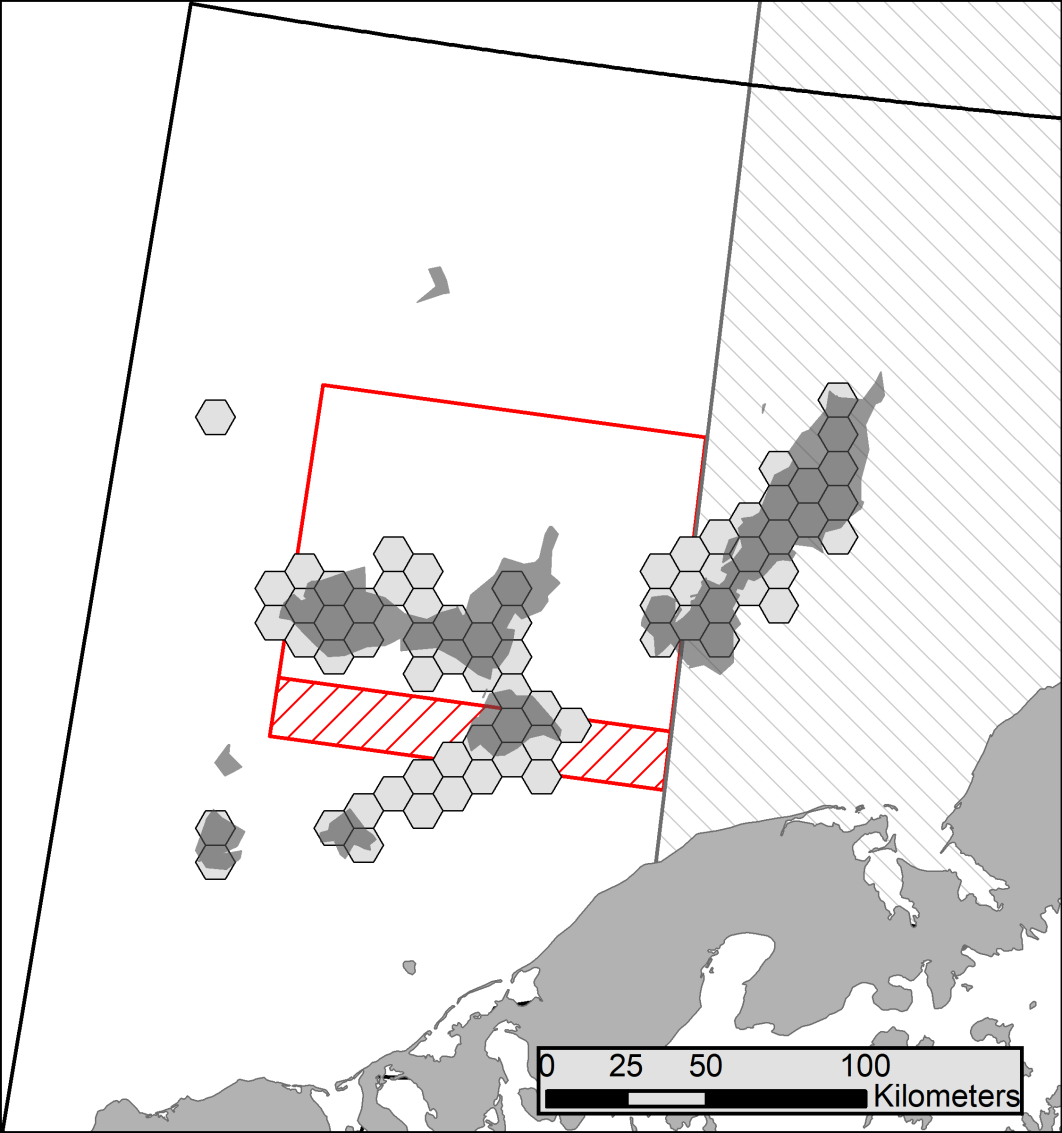
Persistent hot spots and high catch areas for red king crab over 2005–2016. Irregular polygons are hot spots (Getis-Ord, *G*_i_^*^; Fig 4) that persisted in those areas for at least two years and hexagonal polygons had a high crab catch (≥ 20,00 crab caught; Fig 6) for at least two years. Areas with restrictions on trawling are outlined in red or gray and described in Fig 1.

Overall, the patterns in total catch per grid cell (Fig 6) gave similar results to the hot spot analyses (Fig 4). For some years, the hot spots and high catch areas (≥ 20,000 crab/ 100 km^2^) were almost identical (2010 and 2015), but for most years some differences occurred. High catch areas generally fell over hot spot areas, but they often also occurred in areas without hot spots. For example, there are high catch areas in the RKCSS in 2012 and 2013, but this area is not a hot spot in either year. Occasionally, high catch areas even occurred in the same location as cold spots (e.g., 2014). Fig 7 shows hot spots and high catch areas that persisted for at least two years. These two metrics give very similar results, except for the area just south of the RKCSA, which is consistently a high catch area, but not a hot spot.

### Distribution with temperature regime

**Fig 8:**
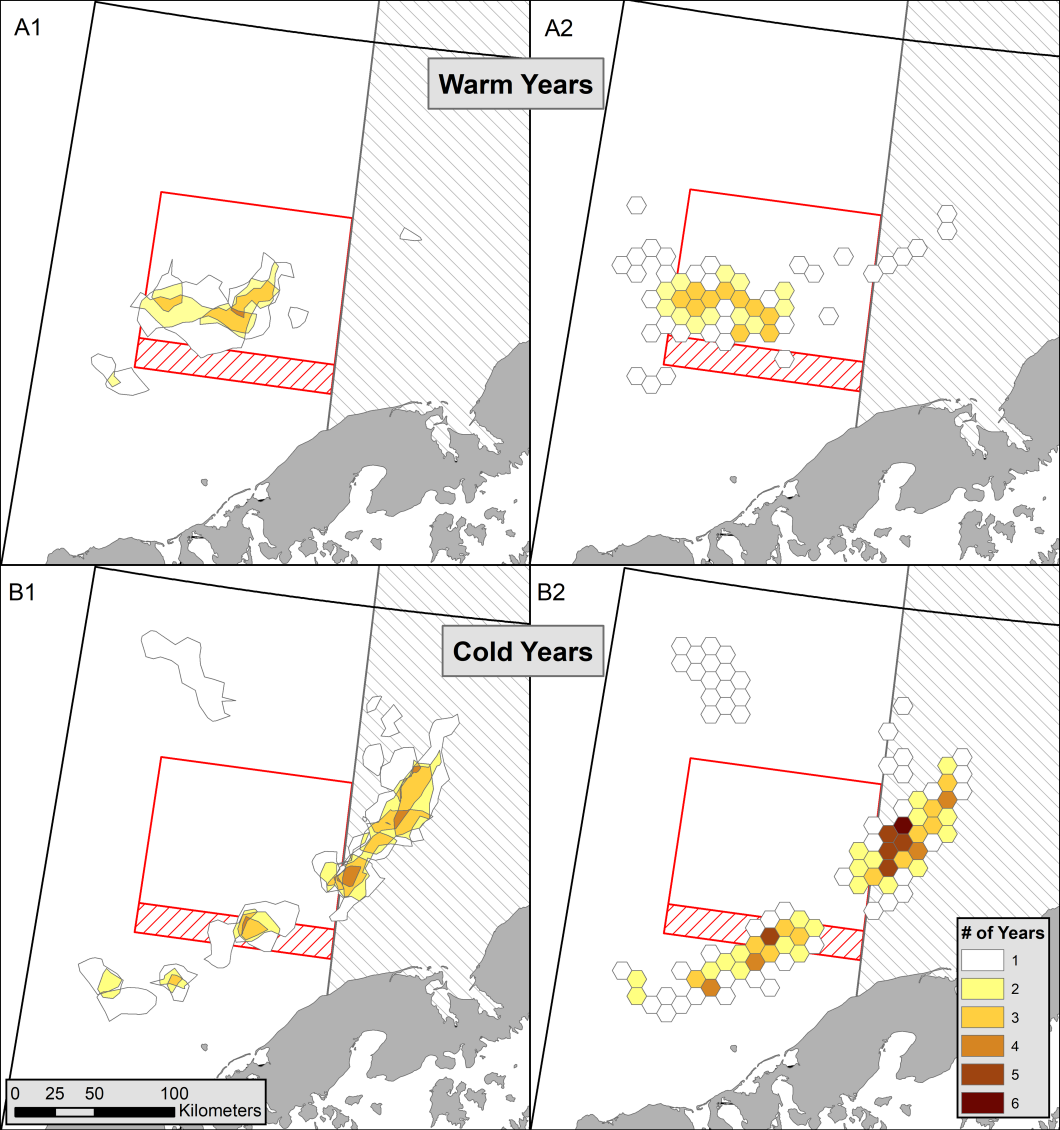
Hot spots and high catch areas for red king crab in warm and cold years. Getis-Ord hot spots, *G*_i_^*^ for catch per unit effort (A1, B1; red areas from Fig 4) and high crab catch areas (A2, B2; red grid cells in Fig 6) for Bristol Bay red king crab using daily fishing logs for A) warm years (2005, 2014–2016) and B) cold years (2007–2013) in the Bering Sea (characterized after Duffy-Anderson (2017) [15]). The legend indicates the number of years a hot spot or high catch area occurred in that location. Areas with restrictions on trawling are outlined in red or gray and described in Fig 1.

In warm and cold years, hot spots occurred in different locations in Bristol Bay (Fig 8). In warm years (2005, 2014–2016), hot spots consistently fell in central Bristol Bay within the RKCSA (Fig 8A). In contrast, in cold years (2007–2013), hot spots occurred in a band along the Alaska Peninsula and further east in Bristol Bay (Fig 8B). The exact locations and intensities of hot spots varied among cold years, but in general they were 1) south of the western section of the RKCSS, 2) in the eastern portion of the RKCSS, and 3) east of the RKCSA in the NBBTCA. In 2006, which was a moderate transition year between previous warm and subsequent cold years, the distribution was different, with a hot spot to the northeast of the RKCSA (Fig 4). This northern hot spot also occurred in 2007 (Fig 4).

### Trawl closure areas

**Fig 9:**
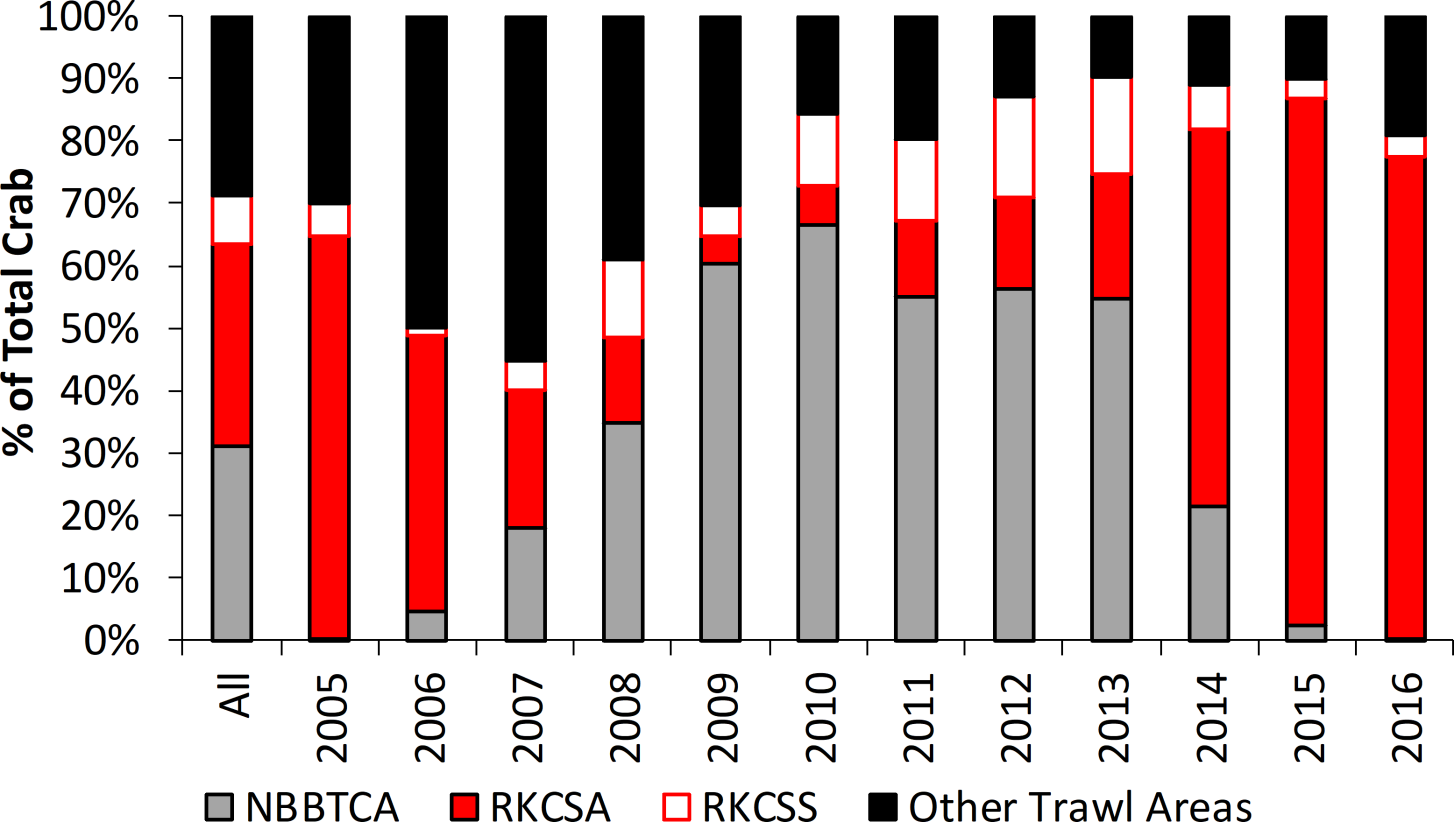
Bristol Bay red king crab catch across protected areas for 2005–2016. Bars show percent of red king crab caught in trawlable areas (RKCSS, Other) and trawl closure areas (NBBTCA, RKCSA) in the Bristol Bay red king crab fishery. NBBTCA = Nearshore Bristol Bay Trawl Closure Area, RKCSA = Red King Crab Savings Area, RKCSS = Red King Crab savings Sub-Area.

Over the 12-year period, 63.4% of the RKC catch occurred in no-trawl zones (Fig 9). On average, 32.2% of crab were caught in the RKCSA, 7.8% in the RKCSS, 31.1% in the NBBTCA, and 28.8% in other areas that can be trawled. In general, more crab were caught in closure areas in warm years than in cold years, with 2006, 2007, and 2008 having the lowest number of crab caught in protected areas.

## Discussion

Here, we displayed the usefulness of DFLs in revealing the spatial distribution of a highly mobile commercial species across Bristol Bay, during a season when survey data are not available. The high correspondence of spatial patterns in crab distributions between DFLs and observer data help validate the accuracy of both datasets. Spatial analyses showed very different patterns in crab distribution depending on temperature regime in the Bering Sea. In warm years, crab were aggregated in a central location within the RKCSA, while in cold years they were concentrated in a band along the Alaska Peninsula. Most crab were harvested within the trawl-closure areas, yet the importance of each closure area varied by year and temperature regime.

### Crab distribution

Autumn distribution data from DFLs and observer reports add a new layer to our previous understanding of RKC distributions from the well-established NMFS summer trawl surveys. Comparing RKC distributions inferred from the NMFS survey and DFLs is challenging because of differences in sampling design and area covered (S3 Fig). The NMFS survey has equal sampling effort across most of the continental shelf, with one trawl per 20 x 20 nautical mile grid cell. In contrast, the total area covered by DFL data is more restricted, but the sampling effort within the localized area is higher, although not uniform. Nonetheless, compared with autumn DFL data, NMFS summer data typically show higher numbers of legal male crab further north and east in Bristol Bay and/or closer to the Alaska Peninsula. The differences between summer and autumn distributions of Bristol Bay RKC show the importance of examining distributions at different times of year, especially when these data inform fishery management decisions.

Hot spot analyses (Figs 4 and 5) and total catch (Fig 6) provided two distinct metrics for examining autumn distribution of RKC in Bristol Bay. Hot spots are defined here as areas with significantly higher CPUE than the annual mean. In contrast, high catch areas are places where large numbers of crab were extracted over the fishing season (≥ 20,000 crab/100 km^2^ grid cell). When a location was a hot spot, but not a high catch area, the CPUE was high, but there was not enough sustained effort in that location to catch at least 20,000 crab/100 km^2^; it is not possible to determine if these locations would have had enough crab to become high catch areas if fishing effort had been higher. High catch areas that were not hot spots had large numbers of crab extracted, but CPUE was not higher than average. These areas are consistently important areas for crab harvest, but they likely do not have large localized aggregations that can produce high CPUE. In general, hot spots and high catch areas overlapped, verifying the importance of these areas as crab habitat using two separate methods (Fig 7). One important area that was consistently a high catch area, but never a hot spot was the region just south of the RKCSS (Figs 4 and 6). These results suggest that crab are abundant in this region, but they are not highly aggregated, causing CPUE to be average, while large numbers of crab are extracted.

Maps of mean CPUE across the 12 study years (Fig 3), emphasize annual variability in CPUE distribution and intensity. Crab abundance, aggregating behavior, and management practices are all possible contributors to annual CPUE. Throughout a fishing season CPUE generally declines as crab are extracted, thus years with a higher exploitation rate would be expected to have a lower mean CPUE. From 2008 to 2011 the exploitation rate of legal RKC was halved (S2 Fig), which could partially explain patterns in CPUE (e.g., 2008–2010 had lower CPUE than 2011–2013; Fig 3 and S2 Fig). In contrast, the steady decline in legal crab abundance, estimated to decline from 12.4 to 9.4 million crab from 2010 to 2016 [21], did not decrease mean CPUE. For example, CPUE was lowest in 2010 (18 crab/pot) when legal crab populations were highest and CPUE was highest (40 crab/pot) in 2016 when crab populations were lowest (S2 Fig). Crab CPUE could also have been affected by temperature; warm years generally had higher CPUE than cold years, thus it is probable that crab formed tighter aggregations in warm years. When CPUE maps (Fig 3) are compared with hot spot maps (Fig 4), similar, but not identical, patterns emerged. CPUE maps help emphasize that hot spots are only hot spots relative to the year in which they occur. For example, an area detected as a hot spot 2010, which had a very low mean CPUE, would not likely be a hot spot if it had occurred in 2016, when mean CPUE was twice as high.

### Temperature effects

Bristol Bay RKC distributions have primarily been studied for female crab around the breeding season in late spring and early summer [3,10,11,33]. Adult females and at least a portion of the adult males migrate to nearshore areas for mating in the spring [10,11,34]. Migrations out of nearshore areas after breeding occur later in colder years [10,11], because cold temperatures delay embryo maturation and mating [35,36]. The extent and location of the Bering Sea cold pool not only affects the timing of these migrations, but is likely responsible for larger inter-annual shifts in spring distribution of female crab [3,10,33,37]. For example, in the 1970s when there was a large cold pool in Bristol Bay, female crab mostly occurred in southwestern Bristol Bay, near Unimak Island (Fig 1). When temperatures warmed in the late 1970s and early 1980s, female crab distributions shifted to central Bristol Bay [3]. Overall, female RKC appear to avoid waters < 2 ^o^C to remain in optimal temperatures for embryo development [10].

The influence of temperature on the distribution of male RKC is poorly understood [10]. During the breeding season, at least a portion of the mature male population must occur at the same nearshore sites as females, and thus be governed by the female’s temperature requirements. However, in the autumn, male RKC are generally not associated with females [38]. DFL data clearly show that legal sized male crab have distinctly different autumn distributions during warm and cold temperature regimes (Fig 8). The influence of temperature on distribution was particularly apparent in 2014, the first warm year in almost a decade, when male crab returned to the location they had inhabited during the previous warm year, 2005 (Figs 4–6). There are few historical data on autumn RKC distributions with which to determine if these patterns hold over longer timescales. At least in some years in the 1960s, the autumn Japanese tangle net grounds were located west of the RKCSA [34,39], where few crab were captured in our study. However, it is unclear if this represents a difference in autumn distribution or timing of the fisheries. For example, in 1967, the so called autumn tangle net fishery occurred from July to September [34], while in our study most crab were caught between October 15^th^ and November 14^th^. In the United States domestic fishery, ADF&G reports catch by large statistical areas. Comparing a very cold year (1999) and a very warm year (1990) in the Bering Sea, the statistical areas are too coarse to determine whether similar distribution patterns hold as described herein; however, in both of these years more crab were caught in northern quadrants of the RKCSA than occurred in any years reported here [40,41]. This discrepancy points to the importance of continued monitoring of the autumn distribution of legal male RKC, as future shifts in distribution are possible.

Although shifts in RKC distribution appear to be related to temperature regime, the mechanism behind the male crab response to these temperature shifts outside the breeding season requires further exploration. RKC can tolerate a wide range of temperatures; over 30 years of summer NMFS trawl surveys in the Bering Sea, RKC were found in areas where bottom temperatures were between -0.8 and 12.8 ^o^C, with an average of 3.2 ^o^C [42]. Bering Sea crab may migrate offshore to encounter cooler waters during warm years [43], or may avoid extremely cold water in years when the cold pool is present in Bristol Bay [3]. We propose the former is more probable for legal male crab during the autumn fishery. Regardless of temperature regime, autumn bottom water temperatures are the warmest that crab experience over the course of a year, due to vertical mixing of warm surface waters in autumn [14]. In a laboratory study, when placed in a thermal gradient, mature male RKC occurred across the gradient (< 1 ^o^C to 14 ^o^C), but they typically avoided temperatures > 4 ^o^C and on average favored waters between 2.7 and 3.0 ^o^C [44]. Adult males did not avoid the coldest waters and occurred equally at 0–2 ^o^C and 2–4 ^o^C water [44]. In years when the cold pool is present in Bristol Bay, water temperatures in the autumn rarely exceed 4 ^o^C at the M2 mooring (Fig 1); however, in warm years bottom temperatures generally exceed 4°C for several months in the autumn [14]. Thus, it is more likely that adult male crab move to avoid warm waters > 4 ^o^C during warm years, rather than avoiding cold waters in cold years. Unfortunately, bottom water temperature is not measured across Bristol Bay in the autumn, so temperature comparisons between areas occupied by crab in warm years and cold years cannot be made.

Regardless of the causes for the observed shift in distribution, there could be consequences for crab living in different habitats. Moving westward, out of Bristol Bay, the sediment becomes finer [45]. In cold years, crab hot spots mostly occur where there is a sandy substrate, while in warm years the substrate in hot spot areas is a mud-sand mixture [45]. Sediment grain size is well known to influence benthic community structure [46,47]. In the southeastern Bering Sea, sediment type can dictate diet and distribution of flatfishes [48]. RKC are generalist predators, feeding on a wide variety of organisms, including molluscs, echinoderms, polychaetes, and cnidarians [49]. The influence of substrate type on the distribution of benthic prey species suggests that changes in crab distribution will cause (or perhaps be driven by) shifts in the composition of their diet. For example, yellowfin sole (*Limanda aspera*) in the southern Bering Sea feed predominantly on non-segmented coelomate worms in mud-sand areas, and bivalves on sandy substrate [48]. RKC diets also appear to shift with prey availability [50]; thus RKC diets likely shift from a clam-based diet when they are living on sand in cold years, to a more worm-based diet when living over finer sediments in warm years. The energetic consequences of this suspected diet shift warrant further investigation, as well as potential competitive interactions with other predators utilizing the same prey base.

### Management implications

Given the differences in crab distribution with temperature regime, observed both in summer (NMFS trawl survey) and autumn (DFL data), it is important to evaluate the effectiveness of fixed closure areas designed to protect crab from trawl fisheries. The RKCSA and NBBTCA were closed to trawling to protect adult crab and juveniles, respectively [8], yet limited data were available to guide the placement of these closures. The NBBTCA was placed in an area known to meet juvenile habitat requirements [51], while the RKCSA was created based on crab abundance from summer trawl surveys and high bycatch of RKC in a few seasons for some of the winter flatfish fisheries [8]. Despite the limited data to support these decisions, in the years directly after implementation of the RKCSA, bycatch rates declined [52] and bycatch has since remained well below the prohibited species catch [20,53]. However, while trawl closures do have clear benefits, the displacement of trawling effort to other locations may cause additional concerns, including increased Pacific halibut (*Hippoglossus stenolepis*) bycatch [52]. These potential negative impacts have motivated an interest in evaluating the effectiveness of trawl closure areas in protecting Bristol Bay RKC.

DFLs yield distribution information in the autumn, just before the start of winter trawl fisheries. Such information should be particularly relevant in evaluating closure areas, relative to the summer survey data. In warm years, 60% of legal male crab were caught by the crab fishery in the RKCSA, while less than 25% were caught there in cold years (Fig 9). In most cold years, over 50% of crab were caught in the NBBTCA. In some cold years, crab also occurred in trawlable waters, both in the RKCSS and in other areas of Zone 1 (Fig 1). These data indicate that crab were caught in high numbers in the closure areas. However, this information describes the distribution of crab during the autumn crab fishery, whereas trawl closures constrain the flatfish fisheries that mostly take place in the winter. If DFL data are to inform the placement of winter trawl closures, additional study is needed to determine the relationship between autumn and winter crab distributions. Toward this end, a few years of fishery independent surveys during autumn and winter would be invaluable.

## Conclusions

DFLs can help elucidate the distribution of Bristol Bay RKC during the autumn, when standard survey data area not available. They provide insights into patterns in crab distribution with temperature regime and can help inform the placement of closure areas to achieve crab conservation objectives. DFLs provide accurate spatial data, given the similarity of results derived from observer and DFL data. Given the many uses of DFL data, it is essential that they become more accessible in the future for other Bering Sea and Aleutian Island crab fisheries (e.g., golden king crab [*Lithodes aequispinus*] crab, Tanner crab [*Chionoecetes bairdi*], snow crab [*Chionoecetes opilio*]). The Bristol Bay RKC fleet has begun to transition from paper to electronic logbooks, which should make these data more readily available to managers and avoid additional human transcriber errors. RKC are highly mobile species, thus the summer survey cannot fully explain their distribution; DFLs are an important tool that can supplement summer surveys, helping to improve fishery management decisions that concern crab distribution.

## Supporting information

S1 Table Bristol Bay red king crab daily fishing log data aggregated by 100 km^2^ grid cells. Pot strings were assigned to grid cells based on their midpoint. Grid cells with fewer than three vessels fishing in them were excluded due to confidentiality restricitons. Latitude and longitude are for the centroid of each hexagonal grid cell, as in Figs 3 and 6 (Alaska Albers Equal Area Conic using North American 1983 Datum). Two different calculations were used for grid cell catch per unit effort (CPUE). CPUE #1 is the total crab caught per cell divided by the total pots per cell, thus strings with more pots contribute more to cell CPUE than strings with few pots. CPUE #2 (mapped in Fig 3) is the mean CPUE of all points (string midpoints) in a cell, thus all strings contribute equally to cell CPUE.
(XLSX)

S1 Fig Comparisons of weighting methods for Getis-Ord hot spot, Gi* analyses. Analyses performed on catch per unit effort data from daily fishing logs in the Bristol Bay red king crab fishery. Analyses were conducted using 5 weighting methods: a distance band (*d*) of 20, 10, and 5 km and a neighbor number (*k*) of 20 and 40. Results shown for two sample years, 2005 and 2008. Areas with restrictions on trawling are outlined in red or gray and described in Fig 1.
(TIF)

S2 Fig Annual catch per unit effort (CPUE) and exploitation rate. CPUE Total is the mean annual CPUE for 2005–2016 using daily fishing log data from the Bristol Bay red king crab fishery, while CPUE 1^st^ 5% is the mean CPUE from the start of the fishery until 5% of the total legal crab in the population were caught each year. Exploitation rate is the percent of legal crab caught each fishing season. Estimates of annual legal crab abundance and exploitation rate were obtained from the 2017 Stock Assessment and Fishery Evaluation Report (scenario 2b) [21].
(PDF)

S3 Fig Autumn versus summer distribution of Bristol Bay red king crab over 2005–2016. Summer data are from the National Marine Fisheries Service (NMFS) trawl survey and autumn data are from daily fishing logs (this study, Fig 4). Both datasets show the catch of legal sized male crab only. NMFS trawls are on a grid system, with crab caught per km^2^: x = no catch, small circle = 1–100 crab, medium circle = 101–500 crab, large circle = 501–1,000 crab, and yellow star = >1,000 crab. A Getis-Ord hot spot analysis was performed on daily fishing log data: red = statistically significant (α < 0.01) high catch per unit effort (CPUE), blue = statistically significant low CPUE, yellow = not statistically different than the mean, and white = areas where no fishing occurred. Areas with restrictions on trawling are outlined in red or gray and described in Fig 1.
(TIF)

## References

[1] Blackford MG. Pioneering a modern small business: Wakefield Seafoods and the Alaskan frontier Industrial development and the social fabric, Vol. 6 Greenwich: JAI Press Inc; 1979.

[2] Kruse GH, Zheng J, Stram DL. Recovery of the Bristol Bay stock of red king crabs under a rebuilding plan. ICES J Mar Sci. 2010;67: 1866–1874. 10.1093/icesjms/fsq136

[3] Loher T, Armstrong DA. Historical changes in the abundance and distribution of ovigerous red king crabs (*Paralithodes camtschaticus*) in Bristol Bay (Alaska), and potential relationship with bottom temperature. Fish Oceanogr. 2005;14: 292–306. 10.1111/j.1365-2419.2005.00337.x

[4] Garber-Yonts B, Lee J. Economic Status Report Summary: BSAI Crab Fisheries, 2016 [Internet]. North Pacific Fishery Management Council 2016 Available: http://www.npfmc.org/wp-content/PDFdocuments/resources/SAFE/CrabSAFE/2016CrabEconomicSAFEappendix.pdf

[5] Fina M. Rationalization of the Bering Sea and Aleutian Islands crab fisheries. Mar Policy. 2005;29: 311–322. 10.1016/j.marpol.2004.05.005

[6] NPFMC. Amendment 18: Fishery Management Plan for Bering Sea/ Aleutian Islands King and Tanner Crabs [Internet]. North Pacific Fishery Management Council Anchorage, AK; 2004 Available: http://alaskafisheries.noaa.gov/sustainablefisheries/amds/amd18_19/ktc18fmp.pdf

[7] ADF&G. 2014–2015 Commercial King and Tanner Crab Fishing Regulations [Internet]. Alaska Department of Fish and Game Juneau, AK; 2014 Available: https://www.adfg.alaska.gov/static/regulations/fishregulations/pdfs/commercial/2015-2017_king_tanner_crab.pdf

[8] NPFMC. Amendment 37: Fishery Management Plan for the Groudfish Fishery of the Bering Sea and Aleutian Islands [Internet]. North Pacific Fishery Management Council Anchorage, AK; 1996 Available: https://alaskafisheries.noaa.gov/sites/default/files/bsaiamd37fmp.pdf

[9] Bright DB. Life histories of the king crab, *Paralithodes camtschatica*, and the “Tanner” crab, *Chionoecetes bairdi*, in Cook Inlet, Alaska. Ph.D. Dissertation. University of Southern California. 1967.

[10] Chilton EA, Foy RJ, Armistead CE. Temperature effects on assessment of red king crab in Bristol Bay, Alaska In: Kruse GH, Eckert GL, Foy RJ, Lipcius RN, Sainte-Marie B, Stram DL, et al, editors. Biology and management of exploited crab populations under climate change. Fairbanks: Alaska Sea Grant College Program, University of Alaska Fairbanks, AK-SG-10-01; 2010 pp. 249–263. doi:https://10.4027/bmespcc.2010

[11] Dew CB. Red king crab mating success, sex ratio, spatial distribution, and abundance estimates as artifacts of survey timing in Bristol Bay, Alaska. N Am J Fish Manag. 2008;28: 1618–1637. 10.1577/M07-038.1

[12] Loher T. Recruitment variability in southeast Bering Sea red king crab (Paralithodes camtschaticus): The roles of early juvenile habitat requirements, spatial population structure, and physical forcing mechanisms. Ph.D. Dissertation. University of Washington. 2001.

[13] Mueter FJ, Litzow MA. Sea ice retreat alters the biogeography of the Bering Sea continental shelf. Ecol Appl. 2008;18: 309–320. 10.1890/07-0564.1 18488598

[14] Stabeno PJ, Kachel NB, Moore SE, Napp JM, Sigler M, Yamaguchi A, et al Comparison of warm and cold years on the southeastern Bering Sea shelf and some implications for the ecosystem. Deep Sea Res Part 2 Top Stud Oceanogr. 2012;65–70: 31–45. 10.1016/j.dsr2.2012.02.020

[15] Duffy-Anderson JT, Stabeno PJ, Siddon EC, Andrews AG, Cooper DW, Eisner LB, et al Return of warm conditions in the southeastern Bering Sea: Phytoplankton—Fish. PLoS One. 2017;12: e0178955 Available: 10.1371/journal.pone.0178955 28658253 PMC5489148

[16] Evans D, Fey M, Foy R, Olson J. The evaluation of adverse impacts from fishing on crab essential fish habitat: NMFS and NPFMC staff discussion paper, Item C-4(c)(1) January 2012 [Internet]. North Pacific Fishery Management Council. 2012. Available: https://www.npfmc.org/wp-content/PDFdocuments/conservation_issues/EFH/BBRKC_EFH212.pdf

[17] Daly B, Armistead C, Foy R. The 2016 Eastern Bering Sea Continental Shelf Bottom Trawl Survey: Results for Commercial Crab Species. U.S. Department of Commerce, NOAA Technical Memorandum NMFS—AFSC-327. 2016. doi:10.7289/V5/TM-AFSC-327

[18] Fitch H, Schwenzfeier M, Baechler B, Hartill T, Salmon M, Deiman M, et al. Annual Management Report for the Commercial and Subsistence Shellfish Fisheries of the Aleutian Islands, Bering Sea and the Westward Region’s Shellfish Observer Program, 2011/12 [Internet]. Alaska Department of Fish and Game, Fishery Management Report No. 12–22. Anchorage, AK; 2014. Available: http://www.adfg.alaska.gov/FedAidPDFs/FMR14-54.pdf

[19] Aydin K, Barbeaux S, Barnard D, Chilton L, Conners C, Dalton M, et al Stock Assessment and Fishery Evaluation Report for the Groundfish Resources of the Bering Sea/ Aleutian Islands Regions [Internet]. North Pacific Fishery Management Council Anchorage, AK; 2016 Available: https://www.afsc.noaa.gov/REFM/Docs/2016/BSAIsafe.php

[20] NPFMC. Bristol Bay red king crab essential fish habitat and bycatch interactions with groundfish fisheries, Item C-1(b) January 2013 [Internet]. North Pacific Fishery Management Council 2013 Available: https://www.npfmc.org/wp-content/PDFdocuments/conservation_issues/EFH/BBRKC_EFH213.pdf

[21] Bush K, Daly B., Dorn M, Eckert G, Foy R, Garber-Yonts B, et al Stock Assessment and Fishery Evaluation Report for the King and Tanner Crab Fisheries of the Bering Sea and Aleutian Islands Regions [Internet]. North Pacific Fishery Management Council Anchorage, AK; 2017 Available: https://www.npfmc.org/safe-stock-assessment-and-fishery-evaluation-reports/

[22] Nelson TA, Boots B. Detecting spatial hot spots in landscape ecology. Ecography (Cop). 2008;31: 556–566. 10.1111/j.0906-7590.2008.05548.x

[23] Boots B. Local measures of spatial association. Écoscience. 2002;9: 168–176. 10.1080/11956860.2002.11682703

[24] O’Sullivan D, Unwin D. Geographic Information Analysis John Wiley & Sons; 2014.

[25] Ord JK, Getis A. Local spatial autocorrelation statistics: distributional issues and an application. Geogr Anal. 2010;27: 286–306. 10.1111/j.1538-4632.1995.tb00912.x

[26] Moran PAP. Notes on continuous stochastic phenomena. Biometrika. 1950;37: 17–23. 10.2307/2332142 15420245

[27] Anselin L. Local indicators of spatial association-LISA. Geogr Anal. 2010;27: 93–115. 10.1111/j.1538-4632.1995.tb00338.x

[28] Cressie N. Geostatistical analysis of spatial data In: Spatial statistics and digital image analysis. Washington D.C: National Academy Press; 1991 pp. 87–108.

[29] Ord JK, Getis A. Testing for local spatial autocorrelation in the presence of global autocorrelation. J Reg Sci. 2001;41: 411–432. 10.1111/0022-4146.00224

[30] Anselin L, Syabri I, Kho Y. GeoDa: an introduction to spatial data analysis. Geogr Anal. 2006;38: 5–22. 10.1111/j.0016-7363.2005.00671.x

[31] Anselin L. GeoDaTM 0.9 User’s Guide [Internet]. Center for Spatially Integrated Social Science. 2003. Available: http://www.unc.edu/~emch/gisph/geoda093.pdf

[32] ESRI. ArcGIS Desktop: Release 10.3 Environmental Systems Research Institute Redlands CA 2014.

[33] Hsu C. Spatial and temporal distribution patterns of female red king crabs in the southeastern Bering Sea. Ph.D. Dissertation. University of Washingtion. 1987.

[34] Takeshita K, Fujita H, Matsuura S. A note on population structure in the eastern Bering Sea adult red king crab, Paralithodes camtschatica. Proceedings of the international symposium on king and Tanner crabs. Fairbanks: Alaska Sea Grant College Program, University of Alaska Fairbanks, AK-SG-90-04; 1990. pp. 427–433. doi:https://docs.lib.noaa.gov/noaa_documents/Sea_Grant_documents/Alaska/Report_90-04.pdf

[35] Shirley TC, Shirley SM, Korn S. Incubation period, molting, and growth of female red king crabs: Effects of temperature. Proceedings of the international symposium on king and Tanner crabs. Fairbanks: Alaska Sea Grant College Program, University of Alaska Fairbanks, AK-SG-90-04; 1990. pp. 51–63. Available: https://docs.lib.noaa.gov/noaa_documents/Sea_Grant_documents/Alaska/Report_90-04.pdf

[36] Otto R., Macintosh RA, Cummiskey PA. Fecundity and other reproductive parameters of female red king crab (*Paralithodes camtschatica*) in Bristol Bay and Norton Sound, Alaska. Proceedings of the international symposium on king and Tanner crabs. Fairbanks: Alaska Sea Grant College Program, University of Alaska Fairbanks, AK-SG-90-04; 1990. pp. 65–90. Available: https://docs.lib.noaa.gov/noaa_documents/Sea_Grant_documents/Alaska/Report_90-04.pdf

[37] Zheng J, Kruse GH. Recruitment variation of eastern Bering Sea crabs: climate-forcing or top-down effects? Prog Oceanogr. 2006;68: 184–204.

[38] Stone RP, Oclair CE, Shirley TC. Aggregating behavior of ovigerous female red king crab, *Paralithodes camtschaticus*, in Auke Bay, Alaska. Can J Fish Aquat Sci. 1993;50: 750–758.

[39] Simpson RR, Shippen HH. Movement and recovery of tagged king crabs in the eastern Bering Sea, 1955–63. Bull No 24, Int North Pacific Fish Comm. 1968;24: 111–123. Available: http://www.npafc.org/new/inpfc/INPFC Bulletin/Bull No.24/Bull24 p111-123 (Simpson + Shippen).pdf

[40] Nippes WE. Westward region shellfish report to the Alaska Board of Fisheries [Internet]. Alaska Department of Fish and Game, Regional Information Report No. 4K91-4. Kodiak, AK; 1991. Available: http://www.adfg.alaska.gov/fedaidpdfs/rir.4k.1991.04.pdf

[41] ADF&G staff. Annual Management Report for the Shellfish Fisheries of the Westward Region, 1999. Alaska Dep Fish Game, Reg Inf Rep No 4K00-55. Kodiak, AK; 2000; Available: http://www.adfg.alaska.gov/FedAidPDFs/RIR.4K.2000.55.pdf

[42] Stevens BG, Lovrich GA. King crabs of the world: species and distributions In: Stevens BG, editor. King crabs of the world, biology and fisheries management. Boca Raton: CRC Press; 2014 pp. 1–30.

[43] Yeung C, McConnaughey RA. Community structure of eastern Bering Sea epibenthic invertebrates from summer bottom-trawl surveys 1982 to 2002. Mar Ecol Prog Ser. 2006;318: 47–62. 10.3354/meps318047

[44] Christiansen JS, Sparboe M, Sæther BS, Siikavuopio SI. Thermal behaviour and the prospect spread of an invasive benthic top predator onto the Euro-Arctic shelves. Divers Distrib. 2015;21: 1004–1013. 10.1111/ddi.12321

[45] Smith KR, McConnaughey RA. Surficial sediments of the eastern Bering Sea continental shelf: EBSSED database documentation [Internet]. U.S. Dep. Commer., NOAA Tech. Memo. NMFS-AFSC-104, 41 p. 1999. Available: https://www.afsc.noaa.gov/Publications/AFSC-TM/NOAA-TM-AFSC-104.pdf

[46] McGonigle C, Collier JS. Interlinking backscatter, grain size and benthic community structure. Estuar Coast Shelf Sci. 2014;147: 123–136. 10.1016/j.ecss.2014.05.025

[47] Wieser W. The effect of grain size on the distribution of small invertebrates inhabiting the beaches of Puget Sound. Limnol Oceanogr. 1959;4: 181–194. 10.4319/lo.1959.4.2.0181

[48] McConnaughey RA, Smith KR. Associations between flatfish abundance and surficial sediments in the eastern Bering Sea. Can J Fish Aquat Sci. 2000;57: 2410–2419. 10.1139/f00-219

[49] Falk-Petersen J, Renaud P, Anisimova N. Establishment and ecosystem effects of the alien invasive red king crab (Paralithodes camtschaticus) in the Barents Sea—A review. ICES J Mar Sci. 2011;68: 479–488. 10.1093/icesjms/fsq192

[50] Sundet JH, Rafter E., Nilssen EM. Sex and seasonal variation in the stomach content of red king crab, *Paralithodes camtschaticus* in the southern Barents Sea. In: Klein JC V., Schram FR, editors. The Biodiversity Crisis and Crustacea: Proceedings of the Fourth International Crustacean Congress, Amsterdam, Netherlands, 20–24 July 1998, Volume 2. Amsterdam: A.A. Balkema Publishers; 2000. pp. 193–200.

[51] Ackley D, Witherell D. Development of a marine habit protection area in Bristol Bay, Alaska Ecosystem approaches for fisheries management. Fairbanks: University of Alaska Sea Grant College Program, AK-SF-99-01; 1999 pp. 511–526. 10.4027/eafm.1999.38

[52] Abbott JK, Haynie AC. What are we protecting? Fisher behavior and the unintended consequences of spatial closures as a fishery management tool. Ecol Appl. 2012;22: 762–777. 10.1890/11-1319.1 22645809

[53] Fissel B, Dalton M, Felthoven R, Garber-Yonts B, Haynie A, Kasperski S, et al Stock Assessment and Fishery Evaluation Report for the Groundfish Fisheries of the Gulf of Alaska and Bering Sea/ Aleutian Islands Area: Economic Status of the Groundfish Fisheries Off Alaska, 2015 [Internet]. North Pacific Fishery Management Council 2016 Available: https://www.afsc.noaa.gov/REFM/Docs/2016/economic.pdf

