# Supplementary figures and images for: Autumn distribution of Bristol Bay red king crab using fishery logbooks
Source: PLoS One. 2018 Jul 20;13(7):e0201190. doi: 10.1371/journal.pone.0201190 (PMC6054397; doi:10.1371/journal.pone.0201190)

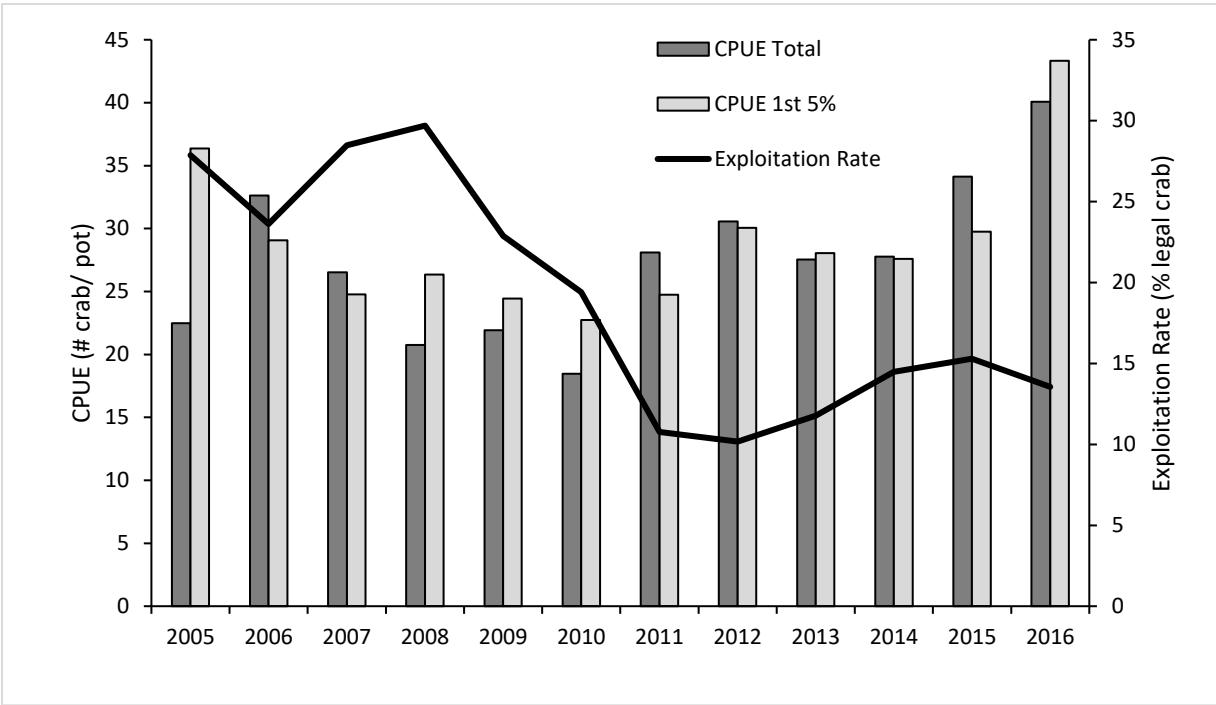

Supplement: S2 Fig — CPUE Total is the mean annual CPUE for 2005–2016 using daily fishing log data from the Bristol Bay red king crab fishery, while CPUE 1st 5% is the mean CPUE from the start of the fishery until 5% of the total legal crab in the population were caught each year. Exploitation rate is the percent of legal crab caught each fishing season. Estimates of annual legal crab abundance and exploitation rate were obtained from the 2017 Stock Assessment and Fishery Evaluation Report (scenario 2b) [21]. (PDF) [file pone.0201190.s003.pdf]
